# Cartilage Oligomeric Matrix Protein in Osteoarthritis and Obesity—Do New Considerations Emerge?

**DOI:** 10.3390/ijms25105263

**Published:** 2024-05-12

**Authors:** Sevdalina Nikolova Lambova, Tsvetelina Batsalova, Dzhemal Moten, Balik Dzhambazov

**Affiliations:** 1 Department of Propaedeutics of Internal Diseases “Prof Dr Anton Mitov”, Faculty of Medicine, Medical University—Plovdiv, 4000 Plovdiv, Bulgaria; sevdalina_n@abv.bg; 2 Department of Rheumatology, MHAT “Sveti Mina”, 4000 Plovdiv, Bulgaria; 3 Department of Developmental Biology, Paisii Hilendarski University of Plovdiv, 4000 Plovdiv, Bulgaria; tsvetelina@uni-plovdiv.bg (T.B.); moten@uni-plovdiv.bg (D.M.)

**Keywords:** COMP, osteoarthritis, BMI, obesity, biomarker, diagnosis

## Abstract

The diagnosis of osteoarthritis (OA) is based on radiological changes that are delayed, along with clinical symptoms. Early and very early diagnosis at the stage of molecular pathology may eventually offer an opportunity for early therapeutic intervention that may retard and prevent future damage. Cartilage oligomeric matrix protein (COMP) is a non-collagenous extracellular matrix protein that promotes the secretion and aggregation of collagen and contributes to the stability of the extracellular matrix. There are contradictory literature data and currently, the parameter is used only for scientific purposes and its significance is not well-determined. The serum level of COMP in patients with metabolic type OA of the knee has not been evaluated. The aim of the study was to analyze serum COMP levels in metabolic knee OA and controls with different BMI. Our results showed that the mean COMP values were significantly higher in the control group (1518.69 ± 232.76 ng/mL) compared to the knee OA patients (1294.58 ± 360.77 ng/mL) (*p* = 0.0012). This may be related to the smaller cartilage volume in OA patients. Additionally, COMP levels negatively correlated with disease duration (*p* = 0.04). The COMP level in knee OA with BMI below 30 kg/m^2^ (*n* = 61, 1304.50 ± 350.60 ng/mL) was higher compared to cases with BMI ≥ 30 kg/m^2^ (*n* = 76, 1286.63 ± 370.86 ng/mL), but the difference was not significant (*p* = 0.68). Whether this finding is related to specific features in the evolution of the metabolic type of knee OA remains to be determined. Interestingly, comparison of COMP levels in the controls with different BMI revealed significantly higher values in overweight and obese individuals (1618.36 ± 203.76 ng/mL in controls with BMI ≥ 25 kg/m^2^, *n* = 18, 1406.61 ± 216.41 ng/mL, *n* = 16; *p* = 0.0092). Whether this finding is associated with increased expression of COMP in the adipose tissue or with more intensive cartilage metabolism in relation to higher biomechanical overload in obese patients, considering the earlier development of metabolic type knee OA as an isolated finding, remains to be determined.

## 1. Introduction

Osteoarthritis (OA) is the most frequent joint disease characterized by degeneration of articular cartilage, subchondral sclerosis, osteophyte formation, and joint space narrowing that leads to structural damage, joint dysfunction and disability [1]. It is a whole-joint disease that is associated with pathology of cartilage, bone, synovium and related structures.

The knee is a weight-bearing joint that is most frequently affected in OA. Moreover, there is increasing evidence that obesity is a contributing factor for its development not only due to the biomechanical overload, but also as a result of the action of systemic mediators, of which adipokines play a leading role, that is suggested to provide the link between obesity and joint pathology. The earlier disease onset of knee OA as an isolated form in obese individuals, as well as the higher level of adipokine, suggest the existence of a distinct phenotype of knee OA, i.e., metabolic type [2]. An association between obesity and OA is related not only to the increased biomechanical overload, but also to the systemic effects of obesity, including low-grade inflammation with increased levels of proinflammatory cytokines (IL-1β, TNF-α, and IL-6) and reactive oxygen species, etc. [3].

Articular cartilage contains chondrocytes and the surrounding extracellular matrix (ECM). The ECM is composed of proteoglycans and collagens (mainly collagen II that binds with collagen XI and IX). It also contains smaller amounts of non-collagenous proteins, such as cartilage oligomeric matrix protein (COMP), which promotes collagen secretion and aggregation and contributes to ECM stability. In mature adult cartilage, chondrocytes are considered to be a functionally inert cell type that maintains ECM homeostasis in a low-turnover state [4]. These characteristics of the chondrocytes are also related to the avascularity of articular cartilage and determine a low potential for recovery of cartilage damage. Thus, in OA, which is the most frequent joint disorder, disease-modifying treatment is currently an enigma.

Cartilage oligomeric matrix protein (COMP) is a non-collagenous ECM glycoprotein of the thrombospondin family known as thrombospondin-5. It is found mainly in the human musculoskeletal system, i.e., in articular cartilage, menisci, ligaments, tendons and synovial membrane. However, new studies confirm the presence of COMP in other tissues as well, such as the vitreous body of the eye, heart, and vascular smooth muscle cells [5]. It has also been confirmed that COMP is expressed in adipose tissue and a positive correlation between its level and BMI has been confirmed [6]. It is also present in the serum and in the synovial fluid, the latter concentration being higher [5], and plays a role in collagen secretion and fibrillogenesis. Additionally, COMP has been confirmed to stimulate chondrocyte proliferation and chondrogenesis. It also contributes to the mechanical strength of tendons. The crucial role of COMP in the structure and function of the musculoskeletal system is confirmed by the development of skeletal dysplasias in COMP gene mutations [7]. Pseudoachondroplasia and multiple epiphyseal dysplasia are chondrodysplasias due to COMP gene mutations with dominant inheritance that are characterized by short stature and early onset of OA [8].

In an in vitro study of cartilage samples obtained from OA patients undergoing knee replacement surgery, Maly et al. [4] studied the expression of COMP and its close family member thrombospondin-4. Non-OA cartilage sections were also evaluated. In healthy cartilage, COMP was uniformly expressed in all layers. In the deep layers, the staining was profound in the interterritorial matrix. In OA, the staining in the superficial zone became weaker, and interterritorial COMP was found to be primarily degraded. Intracellular staining of COMP was detected in the superficial zone, which suggested a process of COMP re-expression. Thrombospondin-4 (TSP-4) was hardly detectable in healthy cartilage. However, a high level of expression was detected in OA, mainly in the middle and deeper zones of articular cartilage vs. predominantly superficial re-expression of COMP [4].

Interestingly, the complex function of COMP is also confirmed by its increased expression in various pathological conditions such as tissue fibrosis, breast and prostate cancer, and cardiomyopathy [7]. In breast cancer, COMP is an emerging prognostic biomarker, and a positive correlation of its level with bone and lung metastases has been found [9].

Biomarkers that facilitate early diagnosis and determination of disease severity and prognosis in knee OA are currently not well defined. In OA, biomarkers that are reliable are currently used as imaging markers [10], i.e., radiography, nuclear magnetic resonance, and ultrasound. The diagnosis of OA is based on radiological changes that are detected late together with clinical symptoms [1,11]. Early and very early diagnosis at the stage of molecular pathology may ultimately offer an opportunity for early therapeutic intervention that may delay and prevent future damage. Biomarkers can also be used to predict disease progression, stratify patients, and monitor disease progression [1,10].

Considerable research has focused on the role of COMP in OA. Higher serum COMP has been reported in knee OA patients compared to controls, but its role in disease assessment is still controversial [10]. There are conflicting literature data and currently, the parameter is used only for scientific purposes and its meaning is not well defined. Bi (2018) performed a meta-analysis of the correlation of serum COMP level with the diagnosis of knee OA [11]. Nine studies and 1694 participants were included. The design of the different studies was variable and included patients with Kellgren–Lawrence radiological stages 1 to 4. A significantly higher serum level of COMP was found in patients with knee OA [11]. The serum level of COMP in patients with metabolic type OA of the knee has not been evaluated. Thus, the aim of this study was to analyze serum COMP levels in metabolic knee OA and controls with different BMI.

## 2. Results

**Figure 1:**
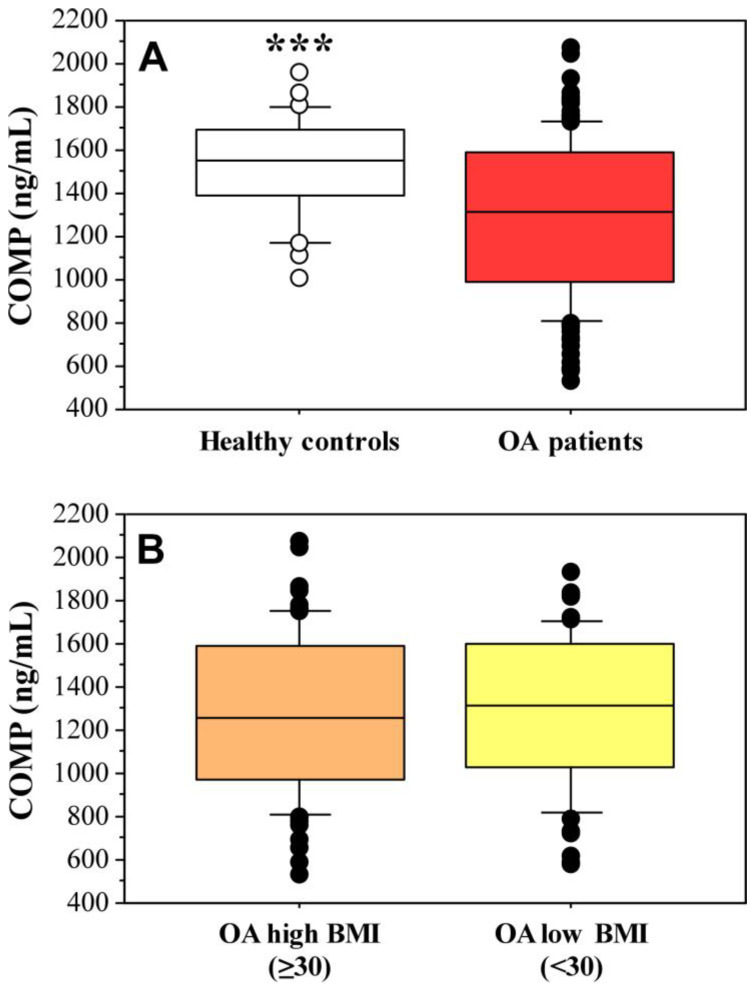
COMP serum levels in healthy controls (*n* = 34) and knee OA (*n* = 137) (**A**), and in knee OA with high BMI (≥30 kg/m^2^, *n* = 77) and low BMI (<30 kg/m^2^, *n* = 60) (**B**). *** *p* = 0.001.

Mean COMP values were significantly higher in the control group (1518.69 ± 232.76 ng/mL) compared to knee OA patients (1294.58 ± 360.77 ng/mL) (*p* = 0.0012, Figure 1A). The mean BMI in patients with knee OA was significantly higher (32.85 ± 8.99 kg/m^2^) compared to the BMI in the control group (25.71 ± 5.12 kg/m^2^). Levels of COMP in knee OA with BMI below 30 kg/m^2^ (*n* = 61, 1304.50 ± 350.60 ng/mL) were higher compared to cases with BMI ≥ 30 kg/m^2^ (*n* = 76, 1286, 63 ± 370.86 ng/mL), but the difference was not significant (*p* = 0.68, Figure 1B).

**Figure 2:**
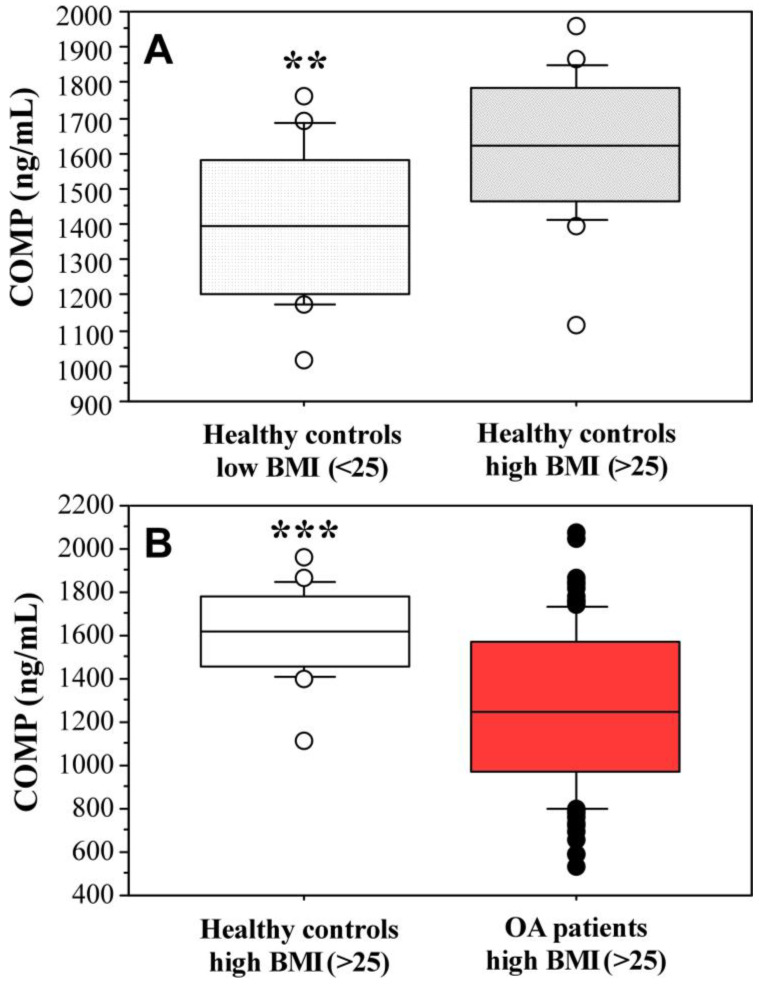
COMP levels in: (**A**) controls with BMI < 25 kg/m^2^ (*n* = 16) and BMI ≥ 25 kg/m^2^ (*n* = 18); (**B**) controls with BMI ≥ 25 kg/m^2^ (*n* = 18) and OA patients with BMI ≥ 25 kg/m^2^ (*n* = 114). ** *p* = 0.009, *** *p* = 0.0002.

Interestingly, comparison of COMP levels in controls with different BMI revealed significantly higher values in overweight and obese individuals (1618.36 ± 203.76 ng/mL in controls with BMI ≥ 25 kg/m^2^, *n* = 18; 1406.61 ± 216.41 ng/mL in controls with BMI < 25 kg/m^2^, *n* = 16; *p* = 0.0092, Figure 2A). Additional analysis that compared COMP levels in osteoarthritic patients and controls with BMI ≥ 25 kg/m^2^ showed significantly higher values (*p* = 0.0002) again in the control group (Figure 2B).

**Figure 3:**
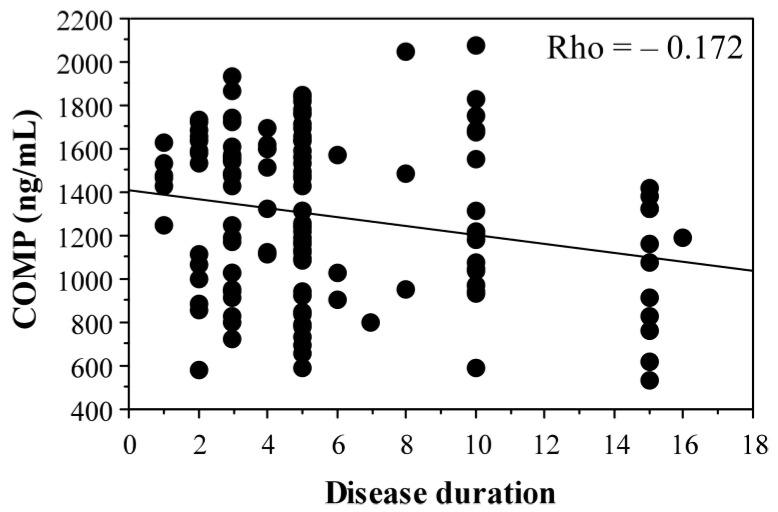
Correlation of serum COMP level in knee OA patients and disease duration.

Of note, COMP levels correlated negatively with disease duration (*p* = 0.04, Figure 3).

**Figure 4:**
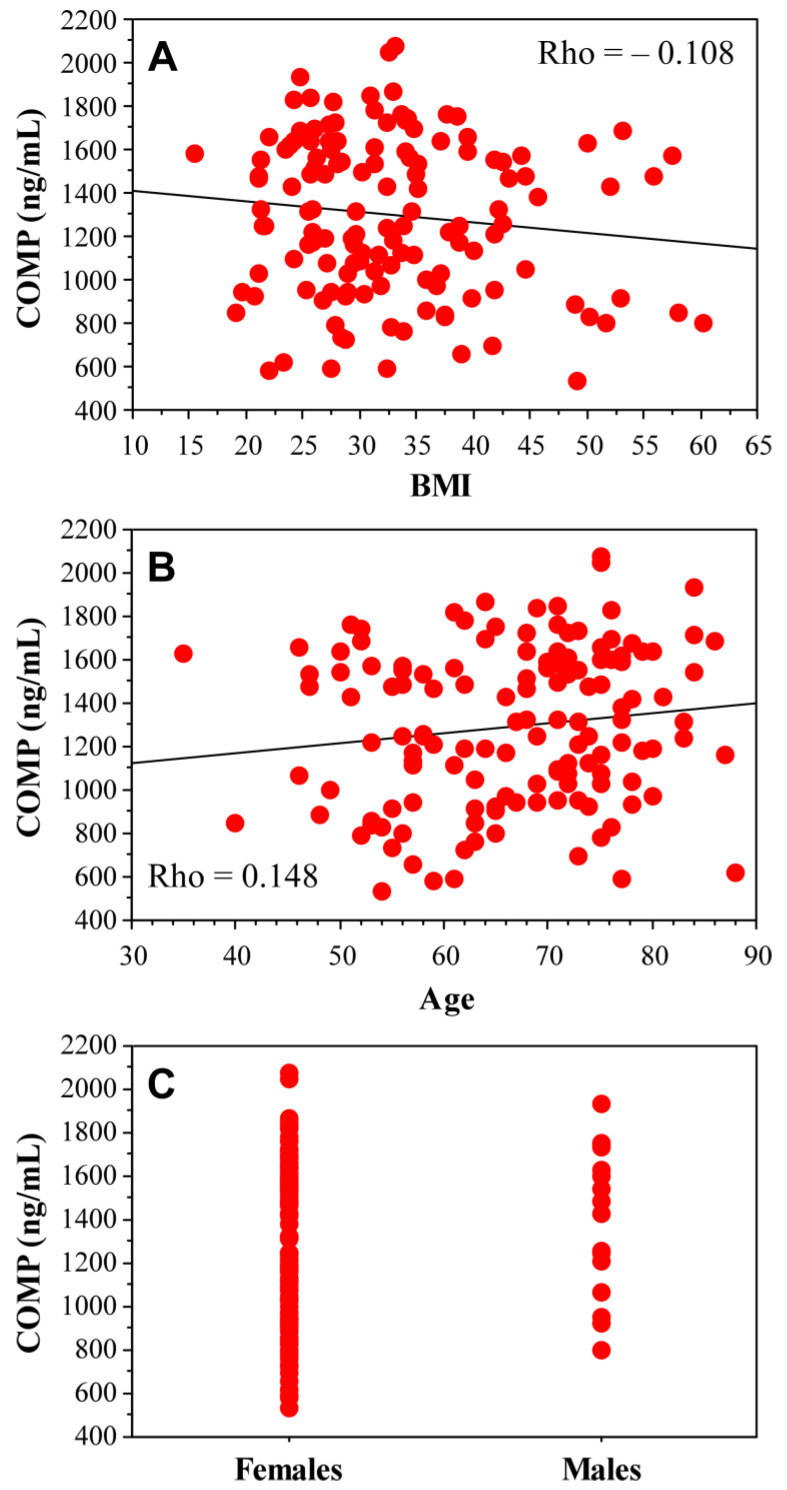
Correlation of serum COMP concentrations with BMI (**A**), age (**B**) and gender (**C**).

In all knee OA patients (*n* = 137), COMP values also did not correlate significantly with the BMI (*p* = 0.2091), age (*p* = 0.0847) and gender (*p* = 0.9206) (Figure 4).

**Figure 5:**
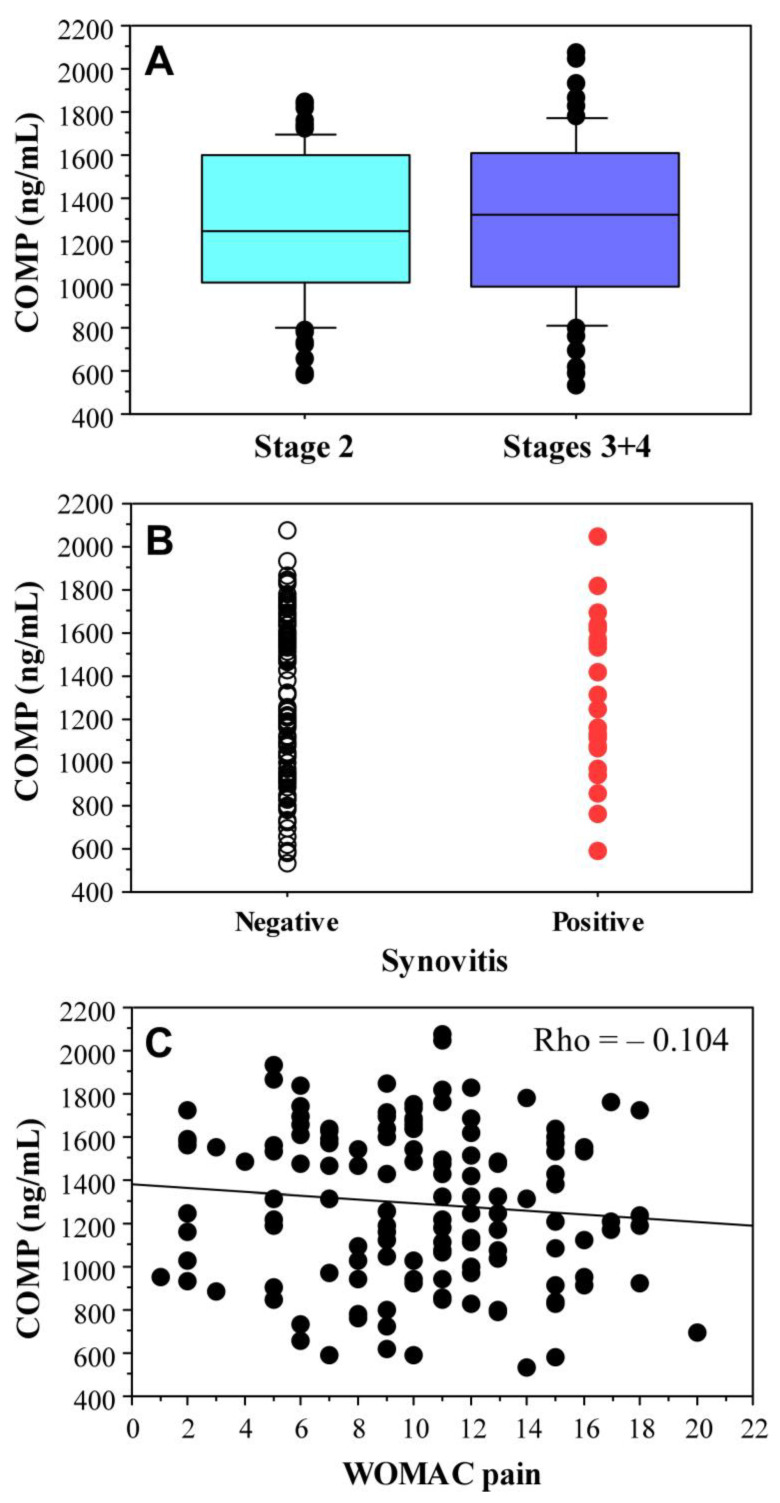
Association between serum COMP level and radiological stage (**A**), presence of ultrasound evidence of synovitis (**B**), and WOMAC pain scale (**C**).

Levels of COMP were not significantly different between radiological stage 2 knee OA (*n* = 75) and those with more advanced changes (stages 3 and 4, *n* = 59) (*p* = 0.70), (Figure 5A). No association was found between COMP level and the presence of synovitis (*p* = 0.80, Figure 5B) and WOMAC pain (*p* = 0.21, Figure 5C).

## 3. Discussion

### 3.1. COMP Levels in Knee OA and in Controls

In the present study, the mean COMP values were significantly higher in the control group (1518.69 ± 232.76 ng/mL) compared to the knee OA patients (1294.58 ± 360.77 ng/mL) (*p* = 0.0012). This may be related to the smaller cartilage volume in OA patients. Additionally, COMP levels negatively correlated with disease duration (*p* = 0.04).

In contrast, Verma et al. [12] found significantly higher levels of COMP in a group of patients with knee OA that also included cases with early OA. However, the decrease in COMP level during the course of the disease was not significantly correlated with radiological grading. These findings were explained by the decrease in the number of chondrocytes in the evolution of OA. Thus, they cannot regenerate the extracellular cartilage matrix [12].

Vilim et al. [13] conducted a prospective 3-year study including 48 patients with symptomatic primary knee OA, grades I–III, according to the Kellgren–Lawrence scale. The authors found that the change in joint space width correlated positively with the serum COMP level at baseline and at the end of the study. Thus, it is suggested that serum COMP may serve as a potential prognostic marker to predict disease progression [13]. A recent meta-analysis [11] looked at the association of serum COMP level with the diagnosis of knee OA. Based on the analysis of nine studies and 1694 participants, the authors concluded that serum COMP levels in knee OA patients were significantly higher. However, the study design was heterogeneous [11]. In a systematic review and meta-analysis, Hao et al. [14] concluded that COMP performed better in differentiating knee OA from control subjects compared to hip OA cases in which CTX-II (C-terminal cross-linking telopeptide of collagen type II) was a better biomarker for knee OA [14]. At present, the exact role of serum COMP as a diagnostic marker in knee OA is still not well defined.

It should be noted that there are different sensitivity methods used to measure COMP. Some monoclonal antibodies used in laboratory diagnostics have been shown to preferentially recognize COMP degradation products and others the intact COMP molecule [15].

### 3.2. COMP Levels in Knee OA and Controls with Different BMI

In our study, COMP levels in knee OA with BMI below 30 kg/m^2^ (*n* = 61, 1304.50 ± 350.60 ng/mL) were higher compared to cases with BMI ≥ 30 kg/m^2^ (*n* = 76, 1286, 63 ± 370.86 ng/mL), but the difference was not significant (*p* = 0.68). Whether this finding is associated with specific characteristics in the evolution of the metabolic type of knee OA remains to be determined.

The COMP protein has traditionally been studied in bone and cartilage. However, COMP is also expressed in adipose tissue. Denton et al. [6] evaluated the presence of differential expression of COMP in abdominal and gluteal adipose tissue, as well as the relationship of serum COMP with the amount of adipose tissue and its distribution in the body. Examination of subcutaneous abdominal and gluteal adipose tissue biopsies showed a 3-fold higher expression of COMP in gluteal adipose tissue, which co-localized with collagen-1. Circulating levels of COMP were positively associated with BMI but not with adipose tissue distribution (android/gynoid adipose tissue ratio) assessed by dual X-ray absorptiometry [6].

Interestingly, comparison of COMP levels in non-OA controls with different BMI in the present study revealed significantly higher values in overweight and obese individuals (1618.36 ± 203.75 ng/mL in controls with BMI ≥ 25 kg/m^2^, *n* = 18, 1406.61 ± 216.41 ng/mL in controls with BMI < 25 kg/m^2^, *n* = 16; *p* = 0.0092). Whether this finding is related to increased expression of COMP in adipose tissue or to more intensive cartilage metabolism in relation to higher biomechanical overload in obese patients, considering the earlier development of metabolic type of knee OA as an isolated finding [2], remains to be determined. The existence of different isoforms of COMP should also be considered.

Higher COMP levels in control subjects indicate that the decrease is probably primarily related to the osteoarthritic process. Moreover, the BMI in controls was lower compared with the patients in OA. However, higher values in overweight and obese controls vs. those with normal BMI also correlate with the new literature data about the expression of COMP in adipose tissue.

### 3.3. COMP Levels and Its Relation to Physical Activity

Furthermore, it should be considered that COMP levels are influenced by physical activity, which is expected to be lower in patients with knee OA. In 44 sedentary healthy men, Celik et al. [16] noted that moderate walking increased the serum concentration of COMP. After 30 min of walking activity and a subsequent recovery period (rest on a chair for 30 min), a significant decrease in serum COMP concentration was observed [16,17]. Mündermann et al. [18] had similar observations and also found an immediate increase in COMP level immediately after a walking exercise in 10 physically active adults who had no history of lower extremity injuries and were pain-free for at least 6 months prior to the study [18]. Furthermore, this phenomenon has also been observed in patients with knee OA. Andersson et al. [19] studied patients with symptomatic radiographically confirmed OA of the knee, unilateral or bilateral Kellgren–Lawrence grade 3 or higher, aged between 36 and 65 years. A significant increase in COMP level was detected after 60 min of exercise [19]. Considering these data, lower physical activity related to pain in knee OA may explain lower COMP levels in osteoarthritis patients.

### 3.4. COMP Levels in the Synovial Fluid in Knee OA

COMP has also been evaluated in the synovial fluid from knee OA patients. It has been suggested that serum levels of COMP may vary depending on its metabolism and clearance. In addition, factors unrelated to musculoskeletal pathology may alter the serum COMP level, such as age, sex, or diurnal variation. Arrelano et al. [20] compared COMP levels in serum and synovial fluid in 229 participants (124 with primary knee OA and 105 healthy controls). 77 subjects had only synovial fluid samples, 78 had only serum samples, and 74 subjects had both serum and synovial fluid analyzed. Total COMP levels in knee OA patients and controls were significantly higher in synovial fluid (676.6 ng/mL) compared to serum (257.5 ng/mL). The difference between synovial fluid and serum concentrations was significant for both groups, i.e., knee OA patients and healthy controls [20]. El-Arman et al. [21] also found a higher level of synovial fluid compared to serum COMP in 66 patients with primary knee OA with effusion [21]. Local production of COMP by joint tissues was proposed as a possible explanation for this finding, as was the renal clearance of serum COMP [20].

### 3.5. Association between Serum COMP Levels with Age and Gender

Serum COMP levels showed no correlation with age and gender in the present study. Clark et al. [22] evaluated serum COMP concentrations in 143 patients with radiographic knee OA (Kellgren/Lawrence grade > 2) and 148 non-OA controls. Similar to the results of the current study, the authors found no gender differences. However, in contrast, they observed an increase in COMP concentration during aging, with the highest concentrations in the >65 age group, which were significantly higher compared to the 45–54 and 55–64 age groups. The study by Clark et al. also found no association between COMP serum and obesity [22].

### 3.6. COMP Serum Levels and WOMAC Pain Score

Previous studies have reported a relationship between serum levels of COMP and the degree of pain assessed by the WOMAC pain index. Our results differ from those reported by Lutfie [23], who found a significant relationship between WOMAC-pain and serum COMP concentration in 146 elderly knee OA patients aged > 60 years with knee OA [23]. Sowers et al. [24] found a statistically significant positive association between serum COMP level and level of pain and stiffness in 72 women with knee OA. Biennial measurements of COMP during a 10-year follow-up period indicated that progressive increases in COMP levels were associated with subsequent worsening of knee OA as determined by radiographic examination and joint stiffness [24]. Similar to the results of the current study, Garnero et al. [25] found no correlation between serum COMP level and WOMAC index (for pain and physical function) in 67 patients with knee OA [25]. Analogous are the results of El-Arman et al., who also found no association between COMP concentration in both serum and synovial fluid and the WOMAC score [21]. In 57 patients with knee OA, Lai et al. also found no association between serum COMP levels and WOMAC score [15]. In a recent analysis on the subtopic about the absence of consensus on the correlation between WOMAC score and COMP level, the need for future larger studies has been underlined. It has been suggested that the potential association may be related to reduced serum levels of cartilage degradation products and less cartilage loss. However, the opposite hypothesis of the lack of correlation between COMP concentrations and the WOMAC index can be explained by the phenomenon that symptomatic relief does not always reflect the level of tissue damage [26]. Furthermore, if serum COMP levels decrease over the course of the disease and with a decrease in cartilage volume, a positive correlation with the WOMAC index cannot be expected.

### 3.7. COMP Serum Levels and Presence of Synovitis

In the present study, no association was observed between serum COMP level and the presence of synovitis. In contrast, Vilim et al. [17] found a significant correlation between serum COMP level and the presence of synovial inflammation in 196 patients with knee OA. The authors suggested that these results may be related to COMP production by the inflamed synovium or compromised clearance [17]. Svetlova et al. also found significantly higher serum COMP levels in 88 patients with early knee OA and synovitis compared with those without ultrasound evidence of synovitis [27]. In 88 patients with primary knee OA, Živanović et al. also measured a significantly higher mean serum concentration of COMP in patients with synovitis compared to cases without synovitis (based on ultrasound examination) [28].

### 3.8. Data about the Use of Serum COMP Levels as a Biomarker in Other Rheumatic Diseases and in Evaluation of Therapeutic Response

The role of serum COMP concentrations has also been analyzed in patients with rheumatoid arthritis. Sakthiswary et al. [29] reported a significantly increased serum level of COMP in 71 patients with rheumatoid arthritis compared to healthy subjects. A positive correlation was found between serum COMP level and disease activity assessed by the DAS 28 index, with disease duration and a negative correlation with knee cartilage thickness assessed by ultrasound. Furthermore, the correlation between serum COMP concentration and the DAS 28 index was comparable to that of commonly used inflammatory markers, i.e., erythrocyte sedimentation rate and C-reactive protein. Thus, it was concluded that serum COMP level may also serve as a biomarker in rheumatoid arthritis due to its association with disease activity and articular cartilage damage [29].

The potential of COMP to serve as a biomarker to assess therapeutic response has also been explored. Lai et al. [15] evaluated serum COMP levels in 33 patients with rheumatoid arthritis treated with TNF-α inhibitors. A significant decrease in serum COMP levels was found in 19 cases that were determined as responders to treatment. Interestingly, no significant difference in COMP level was found in the non-responder group [15]. Kawashiri et al. [30] also detected a significant decrease in serum COMP level in 45 patients with rheumatoid arthritis treated with etanercept for 6 months who achieved DAS28 remission (*n* = 10). A correlation of serum COMP with DAS28-ESR at baseline was observed. Of note, in patients who did not achieve remission (*n* = 35), the COMP level was not significantly different at baseline compared to the level after 6 months. IgM-rheumatoid factor and anti-CCP antibody titer did not change significantly after a 6-month treatment period with etanercept in the remission group [30]. The use of COMP as a biomarker to assess therapeutic response in preclinical and very early stages of knee OA is unknown, given the lack of disease-modifying treatment for OA, the lack of defined criteria for very early diagnosis, and the heterogeneous nature of OA disease. Existence of variations of COMP level in different clinical phenotypes of the knee also deserves attention.

Limitations of the study should be considered. They include the presence of significant differences in BMI and age between osteoarthritic patients and controls. Moreover, future longitudinal studies, including in the early and preclinical stages of OA, would reveal its precise role as a biomarker in knee OA.

## 4. Materials and Methods

### 4.1. Patients and Methods

The study included 137 patients with symptomatic primary knee OA aged between 35 and 88 years (mean age 66 years, 121 women and 16 men). The control group included 34 individuals, including obese patients but without radiographic knee OA (Kellgren–Lawrence = 0). Cases with overweight and obesity but without knee OA were included as controls in order to assess the influence of dysfunctional fat tissue in the absence of joint pathology. However, the selected control group was significantly younger and had lower BMI than the patients with knee OA. This is related to the high frequency of radiographic knee OA in elderly patients and in those with higher BMI, although asymptomatic subjects. All patients underwent clinical examination and antero-posterior radiologic examination of knee joints bilaterally in a standing position. The inclusion criterion for participation in the study was the presence of symptomatic and radiologically confirmed primary knee OA ≥ 2nd radiographic stage according to the Kellgren–Lawrence scale [31]. Exclusion criteria included the presence of various causes of secondary knee OA [32]. All patients had bilateral knee involvement. Patients were radiologically graded 2 to 4 on the Kellgren–Lawrence scale. Patients in whom the radiographic stage was different in the two knees were classified into the more severe stage group. A total of 76 patients had comorbid obesity (BMI ≥ 30 kg/m^2^) and 61 patients had BMI < 30 kg/m^2^. The duration of knee OA was documented based on the available data for radiological confirmation in association with clinical symptoms during previous referrals of the patient.

The presence or absence of OA with other localization was also documented. In 12 cases, primary generalized OA was present [33]. In 89 patients, OA of the knee was combined with OA of the spine of different localization, and in 26 cases, there was evidence of OA of the knee, hip and spine.

Blood was taken after 12 h of participant fasting and after 30 min of rest. Serum COMP levels were measured using a human ELISA kit (MyBioSourse, San Diego, CA, USA) with a sensitivity of 10 pg/mL. All assays were performed according to the manufacturer’s instructions. All patients underwent ultrasound examination of both knees with an MyLab™Sigma ultrasound scanner, B-mode transducer, frequency 4 to 15 MHz (Esaote S.p.A, Genova, Italy). The presence or absence of B-mode synovitis (synovial hypertrophy and effusion) and synovial PD signal have been documented [34,35].

Pain was assessed using the WOMAC LK 3.1 pain scale. The WOMAC pain scale consists of five items: (1) walking on flat ground; (2) going up or down stairs; (3) pain at night while in bed; (4) sitting or lying; and (5) standing upright. All components are scored on a scale of 0–4, i.e., none (0), mild (1), moderate (2), severe (3), and extreme (4). Scores for all items are summed, and possible scores range from 0 to 20 [36].

All subjects gave their informed consent for inclusion in the study. The study was conducted following the Declaration of Helsinki, and approved by the Local Ethical Committee at Paisii Hilendarski University of Plovdiv, Bulgaria (protocol 5 from 10 June 2020).

### 4.2. Statistical Analysis

Data are presented as means ± standard deviation (SD). Statistical analyses were performed by one-way ANOVA, unpaired *t*-test and Spearman’s rank correlation using GraphPad InStat^®^ 3.0 software (GraphPad Software, Inc., San Diego, CA, USA). Differences were considered significant at *p* < 0.05.

## 5. Conclusions

In the current study, which did not include patients with early knee OA, the mean COMP values were significantly higher in the control group compared to patients with knee OA. This may be related to the lower cartilage volume in OA patients, given that COMP levels correlate negatively with disease duration. The level of COMP in knee OA with a BMI below 30 kg/m^2^ was higher compared to cases with a BMI ≥ 30 kg/m^2^, but the difference was not significant. Whether this finding is related to specific features in the evolution of the metabolic type of knee OA remains to be determined. Comparison of COMP levels in non-OA controls with different BMIs in the present study revealed significantly higher values in overweight and obese individuals. Whether this result is related to the increased expression of COMP in adipose tissue or to a more intense metabolism of cartilage in relation to a higher biomechanical overload in obese patients, considering the earlier development of a metabolic type of knee OA as an isolated finding, remains to be determined. The existence of different isoforms of COMP and the improvement of its laboratory measurement should also be considered.

## Data Availability

Data are contained within the article.

## Abbreviations

ANOVA	analysis of variance
Anti-CCP	anti-cyclic citrullinated peptide
BMI	body mass index
COMP	cartilage oligomeric matrix protein
DAS28	disease activity score-28
ECM	extracellular matrix
ELISA	enzyme-linked immunosorbent assay
ESR	erythrocyte sedimentation rate
IgM	immunoglobulin M
IL-1β	interleukin-1 beta
IL-6	interleukin-6
OA	osteoarthritis
TNF-α	tumor necrosis factor alpha
TSP	thrombospondin
WOMAC	Western Ontario and McMaster Universities index
